# Adverse pregnancy outcomes and risk factors in women with primary Sjögren's syndrome: data from a prospective study

**DOI:** 10.3389/frph.2026.1806179

**Published:** 2026-05-28

**Authors:** Yun Zhu, Yi Yu, Ziyi Jin, Yu Wei, Xuebing Feng, Ying Zhang

**Affiliations:** 1 Department of Rheumatology and Immunology, Nanjing Drum Tower Hospital, the Affiliated Hospital of Medical School, Nanjing University, Nanjing, China; 2 Department of Rheumatology and Immunology, Nanjing Drum Tower Hospital Clinical College of Traditional Chinese and Western Medicine, Nanjing University of Chinese Medicine, Nanjing, China

**Keywords:** adverse pregnancy outcomes, anti-U1-ribonucleoprotein antibody positivity, pregnancy, primary sjögren's syndrome, subchorionic hemorrhage

## Abstract

**Objective:**

This study aimed to describe the pregnancy outcomes in patients with primary Sjögren's syndrome (SjD) and identify risk factors for adverse pregnancy outcomes (APO) in a prospective single center study.

**Methods:**

We described the outcomes of 114 pregnant women who fulfilled the established criteria for SjD and analyzed the risk factors for adverse outcomes using multiple factors logistic regression.

**Results:**

The fetal and maternal outcomes in 114 pregnancies were as follows:100 (87.7%) live births, 11(9.6%) miscarriages (first-trimester pregnancy loss), three (2.6%) stillbirths, six (5.3%) cases with intrauterine growth restriction, five (4.1%) preterm deliveries, seven (6.1%) cases with small-for-gestational-age birth weight, and five (4.4%) cases with preeclampsia or eclampsia. Multivariate analysis revealed that age at pregnancy (odds ratio [OR]:1.402, *P* = 0.016), anti-U1-ribonucleoprotein antibody positivity (OR = 3.562, *P* = 0.029), and subchorionic hemorrhage (OR = 7.652, *P* = 0.002) were independent predictors of APO.

**Conclusion:**

In our cohort, women with subchorionic hemorrhage or anti-U1RNP antibody positivity require close monitoring because these factors are associated with a higher risk of APO.

## Introduction

Primary Sjögren's syndrome (SjD) is an autoimmune disease, that is primarily characterized by dry mouth, dry eyes, chronic pain, and fatigue. However, some women of childbearing age are diagnosed with SjD because of infertility and pathological pregnancy. It is worth noting that pregnant women with pSS face unique clinical challenges: anti-SSA/Ro antibodies can cross the placenta, potentially leading to neonatal lupus or congenital heart block (1). An association between SjD and a greater risk of adverse pregnancy outcomes (APOs) has been reported, and the incidences of miscarriages, intrauterine fetal deaths, preterm deliveries, and preeclampsia/eclampsia have been shown to be significantly higher in pregnant women with SjD (2–9). A meta-analysis reported that the risk of adverse pregnancy outcomes in patients with Sjögren's syndrome is 8.5 times that of the general population (10). Further studies are urgently required to address the effects of SjD on pregnancy.

However, most previous studies on pregnancy outcomes in patients with SjD failed to identify factors predictive of APOs. The multicenter, prospective, GR2 (Groupe de Recherche sur la Grossesse et les Maladies Rares), study recruited 108 pregnancies, with 88 pregnancies followed outcomes (six with adverse outcomes), and excluded early miscarriages (<12 weeks) (11). The risk of APOs was not significantly increased in patients with SjD compared with that in the general population. Moreover, antiphospholipid antibody positivity was more frequent among pregnancies with adverse outcomes than among patients with SjD without adverse outcomes (11). Antiphospholipid antibodies, which are the pathogenic antibodies in antiphospholipid syndrome (APS), induce microthrombosis and inflammation in the placenta and are associated with miscarriage in the early stage and APOs in later periods (12). In the present study, we aimed to describe pregnancy outcomes in patients with SjD and identify factors predictive of APOs in a real world study.

## Methods

### Study design

In this single-center prospective study in China, women with SjD in the first trimester were recruited. These women were followed until the pregnancy outcome occurred at the rheumatology and immunology clinic from July 2021 to July 2024. Clinical manifestations and laboratory indicators were recorded in the first, second, and third trimesters. The pregnancy outcome data were collected by telephone interviews or face to face communication. The local ethics committee of Nanjing Drum Tower Hospital approved the study. All participants provided written informed consent. The studies were conducted in accordance with the Declaration of Helsinki.

### Participants

The management of SjD and pregnancy was decided collegially by the rheumatologist and obstetrician. Trimestrial consultation with a SjD specialist was recommended in addition to standard obstetrical follow-up once a month, or more frequently for pregnancies at high risk of adverse outcomes. Women with other connective tissue diseases, such as rheumatoid arthritis, systemic lupus erythematosus (SLE), mixed connective tissue disease (MCTD), antiphospholipid syndrome (APS), defined according to the Sydney classification criteria or “non-criteria” obstetrical antiphospholipid syndrome (NCAPS) (13) were excluded. For diagnosis of SjD, the patients were required to meet the European American consensus criteria for SjD (14). The APO analysis included only singleton pregnancies. Therapeutic abortions were excluded.

### Procedures

At inclusion, the participants' medical history, demographics, clinical and laboratory findings, and treatments were recorded. During pregnancy, laboratory tests, fetal ultrasound findings, treatment modifications, and complications were recorded at each consultation. Lastly, delivery and birth outcomes were analyzed. APOs criteria included embryonic miscarriages, intrauterine fetal death, neonatal death within the first 28 days of life, placental insufficiency (intrauterine growth retardation; preeclampsia or eclampsia), preterm delivery at less than 37 weeks of gestation, and small-for-gestational-age birth weight.

### Statistical analysis

Continuous variables are expressed as median [Inter Quartile Range(IQR)], and qualitative variables are expressed as number (%). Univariate analysis using the logistic model was conducted to identify risk factors for APOs in patients with SjD. Variables that were significant (*p* < 0.05) in the univariate analysis were included in the multivariate model. For variables with missing data, the number of pregnancies with available data was systematically given in the denominator.

## Results

### Characteristics of pregnant women with SjD and pregnancy outcomes

**Figure 1:**
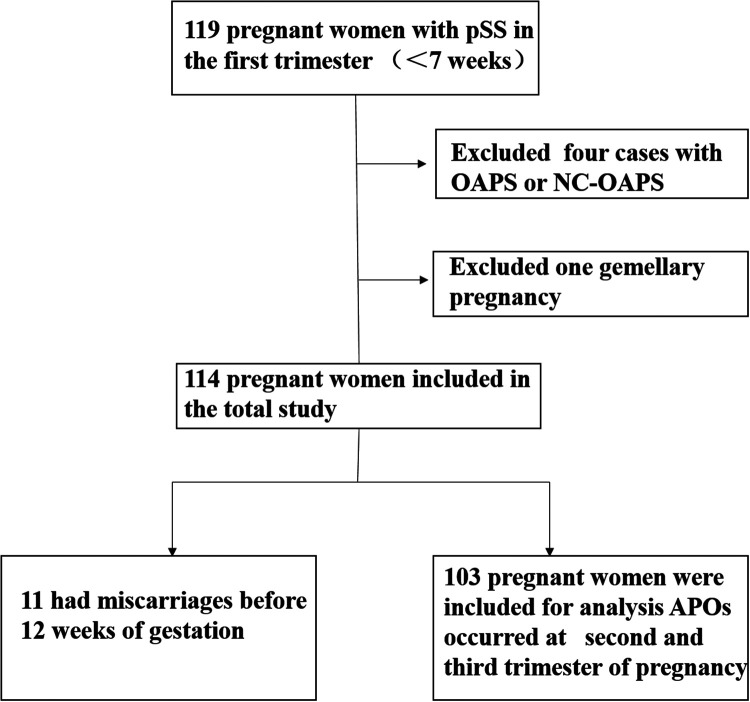
Study profile. APO: Adverse pregnancy outcome, SjD, Primary Sjogren's syndrome; OAPS, obstetric antiphospholipid syndrome; NC-OAPS, “non-criteria” OAPS.

In total, 119 women with SjD in the first trimester were recruited, and four women with APS were excluded. A total of 114 pregnant women with SjD were followed up, and the pregnancy outcome of these patients were analyzed (Figure 1). The demographic, clinical, and laboratory characteristics of the women are summarized in Table 1. The median age at pregnancy onset was 31.5 years (IQR: 30.0, 34.0). A total of 75 (65.8%) of 114 women had a history of previous APOs, 15(13.2%) had three or more consecutive APOs, and 21(18.4%) had APOs occurred after 12 weeks. Notably, 75 (65.8%) of 114 women were diagnosed with SjD because of infertility or pathological pregnancy. Among these 37(32.5%) of 114 women conceived using assisted reproductive technology (ART). Among the 114 pregnancies, all had at least one consultation in each trimester: 31 (29.2%) with dry mouth, 20 (18.9%) with dry eyes, 13 (12.3%) with arthralgia, 16 (15.1%) with rash, and seven (6.5%) with oral ulcer. All patients had low disease activity without organ damage, except for mild thrombocytopenia in four (3.5%) patients, decreased complement 3 in four (3.5%), decreased complement 4 in 26 (22.8%), and increased IgG increased in 30 (26.3%). The anti-SSA, anti-Ro-52, and anti-SSB antibodies were positive in 91(79.8%), 72 (63.2%), and 37 (32.5%) pregnant women, respectively (Table 1). All participants were followed up throughout the pregnancy, during which time they did not experience a SjD flare. Treatments throughout pregnancy included hydroxychloroquine, corticosteroids, low-molecular-weight heparin, and low-dose aspirin. No patient was prescribed a biological therapy.

**Table 1:** Characteristics of the 114 pregnant women with SjD.

Variables	All pregnant women (*n* = 114)
Age at pregnancy onset, years	31.5 [30.0, 34.0]
Body mass index	21.83 [19.70, 23.87]
ART for current pregnant	37 (32.5%)
History	
Hypothyroidism	28 (26.4%)
insulin resistance	8 (7.1%)
APOs	75 (65.8%)
APOs ≥3	15 (13.2%)
APOs occurred after 12 weeks	21 (18.4%)
SjD diagnosed because of production	75 (65.8%)
ESSDAI at early pregnancy	0 [0, 1]
Course (months)	12.0 [4.0, 36.0]
Clinical manifestation at early pregnancy	
Dry mouth	31 (29.2%)
Dry eyes	20 (18.9%)
Arthralgia	13 (12.3%)
Rash	16 (15.1%)
Oral ulcer	7 (6.6%)
Joint stiffness	7 (6.6%)
Headache	6 (5.6%)
Laboratory findings at early pregnancy	
Antinuclear antibody ≥1:320	39 (34.2%)
Anti-SSA positivity	91 (79.8%)
Anti-SSB positivity	37 (32.5%)
Anti-Ro52 positivity	72 (63.2%)
Rheumatoid factor positivity	26 (22.8%)
Anti-U1RNP positivity	12 (10.5%)
≥1 positive antiphospholipid antibodies	10 (9.9%)
Complement 3	
Median	1.18 [1.07, 1.36]
<0.8 g/L	4 (3.5%)
Complement 4	
Median	0.24 [0.19, 0.30]
<0.2 g/L	26 (22.8%)
Immunoglobulin G	
Median	14.35 [11.98, 16.45]
>16 g/L	30 (26.3%)
White blood cells (10^12^)	6.75 [4.93, 8.73]
Lymphocytes (10^12^)	1.65 [1.30, 2.20]
Hemoglobin (g/L)	127.0 [119.5, 136.0]
Hemoglobin <110 g/L	5 (4.4%)
Platelets (10^9^)	212 [175.75, 264.75]
Platelets <100*10^9^	4 (3.5%)
ESR	
Median	16.0 [10.0, 26.0]
ESR increased (>20 mm/h)	31 (27.2%)
Live birth	100 (87.7%)
Adverse pregnancies outcomes	30 (26.3%)
Miscarriages (<12 weeks)	11 (9.6%)
Stillbirth (intrauterine fetal death/neonatal death)	3 (2.6%)
Intrauterine growth retardation	6 (5.3%)
Preeclampsia or eclampsia	5 (4.4%)
Preterm delivery	5 (4.4%)
Small-for-gestational-age birth weight	7 (6.1%)

Data are presented as the median [IQR], *n* (%), *n*/*N* (%), or *p*-values. For variables with missing data, denominators are reported. ART, assisted reproductive technology; APO, Adverse pregnancy outcome; SjD, Primary Sjogren's syndrome; U1RNP, Anti-U1-ribonucleoprotein; ART, Assisted reproductive technology; ESSDAI, European Alliance of Associations for Rheumatology Sjögren's Syndrome Disease Activity Index.

Of the 114 pregnancies, 100 (87.7%) cases had live births, and APOs occurred in 30 cases (26.3%), including 11 embryonic miscarriages, six intrauterine growth retardation, three intrauterine fetal death, five pre-eclampsia/eclampsia, five preterm, seven small-for-gestational-age birth weight (Table 1). We reported one refractory case of fetal death with edema and complete atrioventricular block (CHB). During this pregnancy, this patient was treated with prednisone, HCQ before pregnancy, and intravenous injection of immunoglobulin at 10 weeks. CHB was discovered at 17 weeks, and fetal death occurred at 20 weeks of pregnancy.

### Risk factors associated with APOs in patients with SjD

The logistic proportional hazard model was used to detect risk factors associated with APOs in patients with SjD. Women with adverse outcomes were older than those without adverse outcomes [33.0 (31.0, 34.0) vs. 31.00 (29.0, 33.0), *p* = 0.003, OR: 1.223]. The frequency of SjD diagnosed because of infertility or pathological pregnancy and SjD course did not differ significantly between the two groups. A higher frequency of arthralgia was observed among pregnancies with adverse outcomes compared to those without adverse outcomes [7 (26.0%) vs. 6 (7.6%), *p* = 0.018, OR: 4.285]. Anti-U1-ribonucleoprotein (Anti-U1RNP) positivity was more frequent among pregnancies with adverse outcomes [8 (26.7%)] than among those without adverse outcomes [4 (4.8%); *p* = 0.003, OR: 7.273; Table 2]. The frequency of anti-antiphospholipid antibody positivity was not significant between groups with APO and without APO [3 (10.7%) of 28 vs. 7 (9.6%) of 73, *p* = 0.865, OR: 1.131]. Early pregnancy ultrasound indicated a higher proportion of subchorionic hemorrhage (SCH) in the group with APOs [7 (26.0%) of 30 vs. 7 (8.6%) of 84, *p* = 0.027, OR: 3.700]. Serum immunoglobulin G levels in the third trimester were significantly higher in patients with APOs compared with those without [11.90 (11.1, 17.3) vs. 10.60 (9.51, 13.30), *p* = 0.014, OR: 1.177]. No other variables were enriched among pregnancies with adverse outcomes, including history of APOs, anti-nuclear antibodies titers, anti-SSA or anti-SSB antibody positivity, white blood cells, hemoglobin, platelets, complement 3, complement 4, and disease activity.

**Table 2:** Characteristics in the first, second, and third trimester of pregnancy according to adverse pregnancy outcome status.

Variables	Pregnancies with adverse outcomes (*n* = 30)	Pregnancies without adverse outcomes (*n* = 84)	*P*-value	Odds ratio	95% CI
Age at pregnancy onset	33.0 [31.0, 34.0]	31.00 [29.0, 33.0]	0.003	1.223	1.072–1.395
Hypothyroidism	8 (29.6%)	20 (25.3%)	0.364	1.453	0.648–3.258
Insulin resistance	4 (14.8%)	4 (5.1%)	0.113	3.261	0.755–14.077
Body mass index at pregnancy onset	21.6[19.5, 23.3]	22.34[19.78, 24.18]	0.651	0.976	0.881–1.082
APO ≥3	7 (23.3%)	8 (9.5%)	0.062	2.891	0.947–8.830
ART for current pregnancy	11 (36.7%)	26 (31.0%)	0.542	1.316	0.545–3.182
Course (months)	12.0 [5.0, 36.0]	11.5 [4.00, 34.5]	0.721	1.003	0.986–1.021
ESSDAI at early pregnancy	0 [0, 1]	0 [0, 1]	0.557	0.925	0.523–1.256
SjD diagnosed because of APO and infertility	20 (66.7%)	55 (65.5%)	0.877	1.075	0.429–2.656
Clinical manifestation at early pregnancy					
Dry mouth	11 (40.7%)	20 (25.3%)	0.132	2.028	0.808–5.089
Dry eyes	7 (26.0%)	13 (16.5%)	0.281	1.777	0.624–5.059
Arthralgia	7 (26.0%)	6 (7.6%)	0.018	4.258	1.268–14.101
Laboratory findings in the first trimester					
Anti-nuclear antibody ≥1:320	13 (43.3%)	26 (31.0%)	0.222	1.706	0.724–4.022
Anti-SSA positivity	22 (73.3%)	69 (82.1%)	0.305	0.598	0.224–1.598
Anti-SSB positivity	10 (33.3%)	27 (32.1%)	0.905	1.056	0.435–2.561
Anti-Ro-52 positivity	23 (76.7%)	49 (58.3%)	0.079	2.347	0.907–6.073
Anti-U1RNP positivity	8 (26.7%)	4 (4.8%)	0.003	7.273	2.003–26.411
≥1 Positive antiphospholipid antibody	3 (10.7%)	7 (9.6%)	0.865	1.131	0.271–4.723
Complement 3	1.14 [1.00, 1.37]	1.18 [1.09, 1.36]	0.227	0.298	0.042–2.126
Complement 4	0.25 [0.19,0.30]	0.24 [0.18, 0.30]	0.823	1.938	0.006–23.235
Immunoglobulin G, g/L	15.0[12.85, 17.70]	14.20 [11.40, 16.20]	0.056	1.124	0.997–1.268
White blood cells, 10^12^	5.90[4.40, 8.00]	7.10[5.20, 9.50]	0.194	0.884	0.734–1.065
Hemoglobin, g/L	125.0 [119.0, 136.5]	128.0 [120.0, 137.0]	0.698	0.992	0.951–1.034
Platelets, 10^9^	231.0 [163.0, 267.0]	211.0[183.0, 269.0]	0.418	2.304	0.306–17.373
Subchorionic hemorrhage	7 (26.0%)	7 (8.6%)	0.027	3.700	1.162–11.782
Treatment in the first trimester					
Hydroxychloroquine	29 (96.7%)	78 (92.9%)	0.466	2.231	0.257–19.334
Prednisone	15 (50.0%)	33 (39.3%)	0.309	1.545	0.668–3.576
Low-dose aspirin	14 (46.7%)	45 (53.6%)	0.516	0.758	0.329–1.749
Low molecular weight heparin	18 (60.0%)	41 (48.8%)	0.294	1.573	0.675–3.668
Laboratory findings in the middle trimester					
Complement 3	1.28 [1.125，1.450]	1.370 [1.235，1.470]	0.089	0.111	0.009–1.402
Complement 4	0.25 [0.19, 0.29]	0.26 [0.21, 0.31]	0.573	0.141	0.000–127.823
Immunoglobulin G, g/L	12.8 [10.0-14.0]	11.50 [9.97, 13.73]	0.245	1.097	0.939–1.281
White blood cells (10^12^)	6.80 [5.55，9.65]	8.10 [6.55，10.25]	0.396	0.921	0.762–1.113
Hemoglobin (g/L)	116.5 [109.0，130.5]	115.0 [105.0，125.0]	0.347	1.017	0.982–1.053
Platelets (10^9^)	201.0 [135.0–254.0]	196.0 [155.0–246.0]	0.150	6.500	1.725–15.765
Treatment in the middle trimester					
Hydroxychloroquine	18 (94.7%)	75 (93.8%)	0.871	1.200	0.132–10.915
Prednisone	9 (47.3%)	30 (37.5%)	0.430	1.500	0.547–4.110
Low-dose aspirin	8 (42.1%)	43 (53.8%)	0.364	0.626	0.228–1.720
Low molecular weight heparin	7 (36.8%)	24 (30.0%)	0.564	1.361	0.477–3.880
Laboratory findings in the last trimester					
Complement 3	1.39 (1.22，1.46]	1.37 [1.23, 1.57]	0.800	0.715	0.054–9.535
Complement 4	0.26 [0.21，0.31]	0.26 [0.23，0.32]	0.484	0.093	0.000–17.125
Immunoglobulin G, g/L	11.90 [11.1，17.3]	10.60 [9.50，13.3]	0.014	1.196	1.037–1.380
White blood cells (10^12^)	6.85 (5.10，8.55]	7.90 [6.30，9.65]	0.223	0.856	0.668–1.099
Hemoglobin [g/L)	122.5 [111.5, 137.5]	122.0 [114.0，130.0]	0.978	1.001	0.953–1.050
Platelets	200.0 [92.5，235.5]	184.0 [152.0，229.0]	0.203	0.994	0.985–1.003
Treatment in the last trimester					
Hydroxychloroquine	14 (100%)	69 (94.5%)	0.999	-	-
Prednisone	5 (35.7%)	27 (37.0%)	0.928	0.947	0.287–3.118
Low-dose aspirin	6 (42.9%)	40 (54.8%)	0.832	1.133	0.358–3.590
Low molecular weight heparin	5(35.7%)	24(32.9%)	0.811	1.157	0.350–3.830

Data are presented as the median [IQR], *n* (%), n/N (%), or *p*-values. For variables with missing data, denominators are reported. APO: Adverse pregnancy outcome, ART, Assisted reproductive technology; SjD, Primary Sjogren's syndrome; U1RNP, Anti-U1-ribonucleoprotein; ESSDAI, European Alliance of Associations for Rheumatology Sjögren's Syndrome Disease Activity Index.

To control for confounding factors, this study included age, anti-U1RNP, SCH, IgG concentration, arthralgia and other potential risk factors for pregnancy, such as anti-nuclear antibody, anti-SSA, anti-SSB, anti-Ro-52, antiphospholipid antibody, and a history of three or more adverse pregnancies, as variables in the analysis of risk factors for APOs. Multivariate analysis revealed that age at pregnancy (OR: 1.402, *p* = 0.016), anti-U1RNP positive (OR: 3.562, *p* = 0.029), and SCH (OR: 7.652, *p* = 0.002) were independent predictors of APOs. IgG concentration, arthralgia, anti-nuclear antibody, anti-SSA, anti-SSB, anti-Ro-52, antiphospholipid antibody, and a history of three or more adverse pregnancies were not considered risk factors for APOs.

## Discussion

In this study, age, anti-U1RNP antibodies positive and SCH were found to be risk factors for APO in patients with SjD. SCH refers to the bleeding and accumulation of blood between the chorion and decidua and is caused by the impairment of trophoblast invasion and angiogenic capacity during early placental formation (15). The first trimester SCH has a controversial effect on pregnancy outcome (16–19). In our study, SCH in the first trimester was associated with APOs. Recently, SCH formation was proposed to be closely related to immune dysfunction and assisted reproductive technology (ART) (15, 20). Li et al. observed that the positivity rate of autoantibodies was significantly higher in patients with SCH than in normal pregnant women, and they reported that high titers of antinuclear antibodies were more likely to be a high-risk factor for SCH (21). In our cohort, 14 (12.2%) SCH cases were detected, which was higher than that in the general population (1.87%) (22). Among 14 SCH cases, two (2/19，10.5%) were ANA negativity, and 12 (12/95, 12.6%) were ANA positivity. The rate of SCH decreased in SjD with ANA negativity, but the difference was not significant. Among 14 SCH cases, seven (7/37) were conceived by ART, and seven (7/77) were naturally conceived. The incidence rate of SCH was higher in patients conceived by ART (18.9%) than in those conceived naturally (9.1%), but the difference was not significant.

Little is known about the influence of anti-U1RNP antibodies on pregnancy. A recent interesting observation involved a case of complete CHB and a case of cutaneous neonatal lupus, both born to a mother with positive isolated anti-U1RNP and negative anti-Ro/SSA antibodies (23). The anti-Ro/SS-A and anti-La/SS-B antibodies and, less commonly, the anti-U1RNP antibodies could be transported across the placental barrier from the mother to the fetus. A total of four patients with CHB who were positive for anti-U1RNP and negative for anti-Ro/SS-A and anti-La/SS-B were reported (23–26). Seventeen patients with neonatal lupus born to women positive for anti-U1RNP antibodies in the absence of anti-Ro/SSA were retrieved from the literature search (24). The findings of fetal outcomes in the MCTD cohort with U1RNP positivity showed that the live birth rate was 71.9%, while 18.7% of pregnancies ended in a first-trimester pregnancy loss, 5.4% of cases had IUGR, and 8.9% of pregnancies ended in stillbirth (24). Because more than 10% of the women in this cohort had cardiac involvement and almost one-third of the patients had pulmonary involvement, it was not known whether anti-U1RNP antibodies is an independent risk factor for APOs. Because anti-U1RNP antibodies are a hallmark of vasculitis in connective tissue diseases (27), we hypothesized that anti-U1RNP antibodies might affect the development of placental blood vessels, except for transplacental passage.

Unlike SLE, it is reported that most pregnant women with SjD did not have organ injury, and SjD flares did not contribute to APO (11). Although the presence of maternal anti-Ro/SSA is strongly associated with the development of neonatal cutaneous lupus and fetal complete CHB, the presence of anti-SSA/Ro did not increase the risk of APO in patients with SjD (11). It was reported that antibody (ANA) titer ≥1:160 was a risk factor for APOs in women with antiphospholipid antibody positivity and previous APOs (28). ANA positivity and ANA titers were not associated with APOs in our cohort. A similar result was found in a larger cohort GR2 study (11). The GR2 study recruited women with SjD with pregnancies at 18 weeks of gestation and found that antiphospholipid antibody positivity was more frequent among pregnancies with adverse outcomes [2 (50%) of 4 pregnancies] than among those without adverse outcomes [2 (4%) of 51 pregnancies] (11). However, we enlarged the sample size and reported that the frequency of anti-antiphospholipid antibody positivity was not significant between groups with APO and without APO. In this study no drug, including hydroxychloroquine, steroids, and heparin, was associated with better pregnancy outcomes. In the absence of contraindications or intolerance, patients with aPL or with anti-SSA/SSB positivity were recommended to take hydroxychloroquine for primary and secondary prophylaxis of CHB and low dose aspirin for preeclampsia/IUGR during pregnancy according to EULAR recommendations for the management of women with SLE and aPL (28). The protective role of hydroxychloroquine and low dose aspirin against APOs could not be established in this study, but the interpretability of this finding is limited by the fact that hydroxychloroquine was prescribed to 90% of the included patients.

Our finding might influence clinical practice. Firstly, our results suggest that routine screening for anti-U1RNP antibodies should be considered as part of the pre-pregnancy evaluation for all of the women with SjD. Identifying this serological marker would help stratify patients into higher-risk categories even before conception, thus allowing for targeted counseling and planning. Next, the finding elevates the detection of SCH in early pregnancy to a critical monitoring parameter for SjD patients, particularly those who are anti-U1RNP positive. Its presence should trigger heightened vigilance. Further, we propose that anti-U1RNP antibodies and SCH in the first trimester should classify the pregnancy as high-risk, which often mandates more frequent prenatal visits, specialized care, and access to maternal-fetal medicine specialists. For patients in this high-risk group a protocol of increased surveillance is warranted. This may include more frequent ultrasound assessments to monitor fetal growth, more frequent uterine artery doppler to assess placental perfusion and close monitoring for signs of preeclampsia or placental insufficiency. Finally, while prospective interventional studies are needed, these findings support the following actionable framework, which included screening for anti-U1RNP antibodies before conception, performing a detailed early ultrasound with specific attention to detecting SCH, and increasing the frequency of prenatal monitoring based on the combined risk stratification. In summary, these findings suggest the integration of a new serologic test (anti-U1RNP antibodies) into pre-pregnancy counseling, redefining the clinical significance of SCH within the SjD population, and enabling earlier, more personalized, and potentially more effective risk-stratified care.

This study has some limitations. First, our study recruited population at high risk for APOs, and the odds ratios for the risk factors may be overestimated. Moreover, most patients had low disease activity without severe organ damage. However, this is counterbalanced by the fact that patients with high disease activity were advised to avoid pregnancy. Finally, treatments before and during pregnancy were based on the physician's judgment and experience. Further studies are urgently required to address the potential role of hydroxychloroquine and other drugs in the treatment of SjD.

## Conclusion

In conclusion, our results show that pregnancies in women with SjD have worse prognoses for the mother and fetus. Women should be screened for anti-U1RNP antibodies before conception and SCH in the first trimester, and those who have positive results should be closely monitored because these factors might be associated with a higher risk of complications.

## Data Availability

The datasets presented in this article are not readily available because All data are presented in this article. Requests to access the datasets should be directed to 15951951745@163.com.
